# Efficient Removal of Mercury Ions Stabilized by Gold Solution Using Chitosan–Guar Gum Polymer Blend in Basic Media

**DOI:** 10.3390/polym17070985

**Published:** 2025-04-04

**Authors:** Azwifunimunwe Tshikovhi, Shivani B. Mishra, Ajay K. Mishra, Mokgaotsa J. Mochane, Tshwafo E. Motaung

**Affiliations:** 1 Department of Chemistry, College of Science, Engineering and Technology, University of South Africa, Johannesburg 1709, South Africa; mochamj@unisa.ac.za; 2 Academy of Nanotechnology and Waste Water Innovations, Johannesburg 2007, South Africa; shivani@anwwi.com (S.B.M.); amishra@uwc.ac.za (A.K.M.); 3 Department of Chemistry, Vaal University of Technology, Private Bag X021, Vanderbijlpark 1900, South Africa; 4 Department of Chemistry, Durban University of Technology, Steve Biko Road, Durban 4001, South Africa; 5 Department of Chemistry, University of Western Cape, Bellville 7535, South Africa; 6 Department of Chemistry, Sefako Makgatho Health Science University, P.O. Box 94, Medunsa 0204, South Africa; tshwafo.motaung@smu.ac.za

**Keywords:** adsorption, mercury, chitosan, guar gum, polymer blend

## Abstract

The highly efficient removal of mercury metal ions at a higher pH (basic media) is barely reported in the literature. In this study, we developed a novel adsorbent by blending chitosan with guar gum, designed to effectively remove mercury ions from basic media by stabilizing them with a gold (Au^3^⁺) solution. The FTIR confirmed the compatibility of chitosan and guar gum through hydrogen bonding. The morphology of the blend exhibited an amorphous and porous structure. A mesoporous structure with a surface area, volume, and diameter of 11.843 (m^2^/g), 0.184 (cm^2^/g), and 17.072 nm, respectively, was confirmed by BET. The adsorption behavior was analyzed using isotherms and kinetics models, which best fitted with the pseudo-second-order kinetic model and Freundlich adsorption isotherm model, respectively. The adsorbent was shown to be an excellent candidate for the removal of mercury ions in water, with an adsorption efficiency of 92% at pH 12 in 60 min and a maximum adsorption capacity of 370.37 (mg/g).

## 1. Introduction

Water pollution by heavy metals such as mercury, cadmium, selenium, and lead is of serious concern due to their harmful impacts on both the environment and humans [1,2]. In particular, mercury pollution associated with various industrial and natural activities is categorized as one of the most toxic metals [3]. For drinking water, the World Health Organization (WHO) recommends a maximum permissible limit of 1 ppb of mercury [4]. Mercury exposure is highly toxic, even at low levels, resulting in irreversible effects on the brain, central nervous system, lungs, kidneys, and so forth [5]. Hence, developing an efficient method for the removal of mercury ions from water is crucial for great welfare and to meet required safety requirements [6,7]. Several techniques have been used for the removal of mercury ions in water, which include precipitation, adsorption, reverse osmosis, and coagulation [8,9]. However, some of these methods have shortcomings, such as being very expensive, having low removal efficiencies, and requiring further treatments [10]. Among these techniques, adsorption has proven to be the most efficient technique for the removal of mercury ions from water [11]. This is attributed to its ease of application, effectiveness at extremely low concentrations, and the availability of a wide range of adsorbents like polymer composites, nanoparticles, and active carbons [12,13,14]. Great attention has been drawn to polysaccharide polymers due to their abundance in nature, cost-effectiveness, renewability, and hydrophilic nature. Chitosan (CS) and its derivatives as a bio-adsorbent for wastewater have been broadly investigated by most researchers. CS (Poly-β-(1, 4)-D-glucosamine) is the 2nd most abundant natural polymer after cellulose in the world [15,16]. It is derived from the de-acylation of chitin, a component of crustaceans such as crabs, squid, and shrimp [17,18]. It is known to be non-toxic, biodegradable, and biocompatible, thus making it a good material for biological applications [19,20]. The presence of amino (-NH_2_) and hydroxyl (-OH) functional groups, which act as binding sites, have made CS a good adsorbent for heavy metals [21,22]. Regardless of its great properties, CS is very sensitive to pH. Thus, depending on the pH values, it can dissolve or agglomerate in solution [23,24]. Numerous methods, such as cross-linking, pretreatment, functionalization, and so forth, have been reported to overcome this challenge [25]. However, they tend to alter the material’s properties, resulting in a decrease in adsorption efficiency [26]. Polymer blending with guar gum has been proposed to be the best solution. Guar gum (GG) is a naturally found polymer extracted from the seeds of the Cyamopsis tetragonolobus guar gum plant [27,28], comprising a mannose backbone and a galactose side chain in an average 1:2 ratio [29,30]. It is nontoxic, biodegradable, and hydrophilic [31]. Therefore, it has the potential to blend well with chitosan, resulting in a new material with improved properties, leading to a high adsorption efficiency. Different authors have reported the use of chitosan and guar gum adsorbents for the removal of mercury ions from water. Zhang and others used a chitosan/cellulose biocomposite sponge to effectively and selectively remove mercury ions from water at an optimum pH of 5.5 [32]. Another study by Allouche et al. focused on efficiently removing mercury ions using hybrid cellulose fiber/chitosan foam composites at pH 4 [33]. In a separate study, Gihar and colleagues used acrylamide- and acrylic acid-grafted guar gum as an adsorbent for mercury ions at pH 6 [34]. All of these studies reported the efficient removal of mercury ions at lower pH values. However, there has been no study on the use of chitosan and guar gum composites or blends at a basic pH. Mercury ion removal in basic media is necessary, as it offers an economical and effective solution by cutting down on treatment time and the need for more chemicals.

In this study, we report the blending of chitosan and guar gum by physical solution blending for the highly efficient removal of Hg ^2+^ ions at a higher basic media (pH 12). FTIR, SEM-EDS, and XRD characterization techniques characterized the prepared CS:GG polymer blend. Mercury is known to be unstable, and so, in this work, mercury was stabilized with a gold solution to improve its instability. Gold Au^3+^ is similar to mercury in both mass and ionization energy; thus, a gold ion acts as a strong oxidizing agent that converts/maintains Hg as mercuric ions, which remain in solution [35]. The batch adsorption behavior of mercury ions onto the CS-GG polymer blend will focus on parameters such as initial concentration, pH, adsorbent dosage, and contact time.

## 2. Materials and Methods

### 2.1. Materials

Chitosan (medium molecular weight, deacylation degree of ≥75%), guar gum, acetic acid (CH_3_COOH), hydrochloric acid (HCl), sodium hydroxide (NaOH), and ethylenediaminetetraacetic acid (EDTA) were obtained from Sigma-Aldrich. All chemicals used in this study were analytical grade. All experiments were conducted using deionized water.

### 2.2. Methods

#### Preparation of Polymer Blend

To prepare the Chitosan–Guar gum (CS-GG) polymer blend, chitosan powder was first dissolved in a 2% (*v/v*) acetic acid solution with constant stirring at room temperature. For the guar gum solution, guar gum powder was dissolved in distilled water at room temperature. The two solutions were mixed by physical solution blending at different compositions (weight %) and freeze-dried overnight to obtain the CS-GG blend. Different compositions (wt %) of chitosan and guar gum were prepared, and the blend composition of 75:25 was selected for this study. The weight composition was calculated using the formula in Equation (1).

$$Wt\;\%=\left(mass\;of\;polymer/total\;mass\right)\times 100$$

(1) Mass of polymer=(Wt %/100 )×total mass

### 2.3. Instrumentation

The polymer blend’s morphology and chemical composition were investigated by the use of a JSM-IT 300 instrument (Jeol, Akishima, Japan) and scanning electron microscopy (SEM) equipped with energy dispersive spectroscopy (EDS). A Bruker D8 (Bruker, Billerica, MA, USA) was used to obtain X-ray diffraction (XRD) patterns. To study the two polymers’ compatibility, a Perkin-Elmer PE 1600 FTIR spectrophotometer (Perkin Elmer, Waltham, MA, USA) was used for Fourier-Transform Infrared Spectroscopy (FTIR) analysis in the 400–4500 cm^−1^ range. A Brunauer-Emmett-Teller (BET) QuantaChrome Autosorb IQ3 (Anton Paar, Graz, Austria) was used to measure the CS-GG’s surface area, pore volume, and pore diameter. To determine the concentration of mercury ions in a solution, inductively coupled plasma optical emission spectrometry (ICP-OES, Agilent 720 series, Agilent Technologies, Santa Clara, CA, USA) was employed.

### 2.4. Adsorption Studies

#### 2.4.1. Batch Equilibrium Studies

To obtain a stock solution of Hg^2+^, a known amount of mercuric chloride (HgCl_2_) was dissolved in a solution of gold (Au^3+^). A diluted solution of 1 M HCl or NaOH was used to adjust the pH of the metal ion solution. At room temperature, the samples were shaken for a certain period using a mechanical shaker. After the process, the adsorbent was filtered out, and inductively coupled plasma optical emission spectrometry (ICP-OES) was used to determine the concentration of mercury ions. Batch adsorption experiments were conducted as a function of time, dosage, concentration, and pH. This was carried out with one parameter changed at a time while keeping the other constant. Each experiment was carried out in three runs, and calculations were then made using the final mean values.

The following equations were used to calculate the quantity of metal ions adsorbed (*q_e_*) and the removal percentage (%R):

(2) $$q_{e}=\frac{(C_{0}-C_{e})\;V}{m}$$

(3) $$\%R=\frac{\left(C_{0}-C_{e}\right)}{C_{0}}\times 100$$

in which *q_e_* is the quantity of pollutant adsorbed (mg/g), *C_0_* and *C_e_* are the initial and equilibrium concentrations of the metal pollutant (mg/L), V is the solution’s volume (L), and m is the adsorbent’s mass (g).

#### 2.4.2. Influence of the Co-Existing Heavy Metal Ions

The CS-GG blend’s selectivity on Hg^2+^ ions was evaluated using a solution of mercury (Hg^2+^), lead (Pb^2+^), and cadmium (Cd^2+^) metal ions. The solution was agitated for 60 min at room temperature with a mechanical shaker. Following filtering out the adsorbent at the end of the experiment, ICP-OES was used to measure the quantity of mercury, lead, and cadmium ions.

#### 2.4.3. Batch Kinetic Studies

Kinetic data were evaluated by varying the contact time from 5 to 120 min. The contact time was adjusted between 5 and 120 min to analyze kinetic data. The kinetic tests concluded with an initial concentration of 20 mg/L, a dosage of 40 mg at pH 12, and an agitation speed of 180 rpm. The study was conducted at room temperature.

#### 2.4.4. Desorption and Regeneration Studies

Following the removal of the unadsorbed Hg^2+^ ions through several washes with deionized water, the adsorbed Hg^2+^ ions on the polymer blend were desorbed using 0.1 M EDTA and NaOH desorbing agents. The regenerated CS-GG blend was used in the subsequent adsorption cycle. Five repeats of the adsorption–desorption cycles were performed to assess the adsorbent’s reusability.

## 3. Results and Discussions

### 3.1. Characterisations of CS-GG Polymer Blend

**Figure 1:**
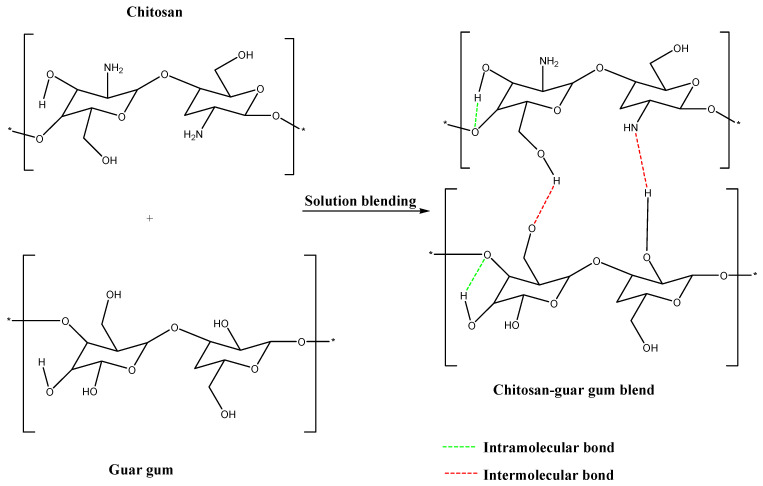
Schematic illustration of chitosan and guar gum blend via hydrogen bonding. * indicates repeating polymer units.

**Figure 2:**
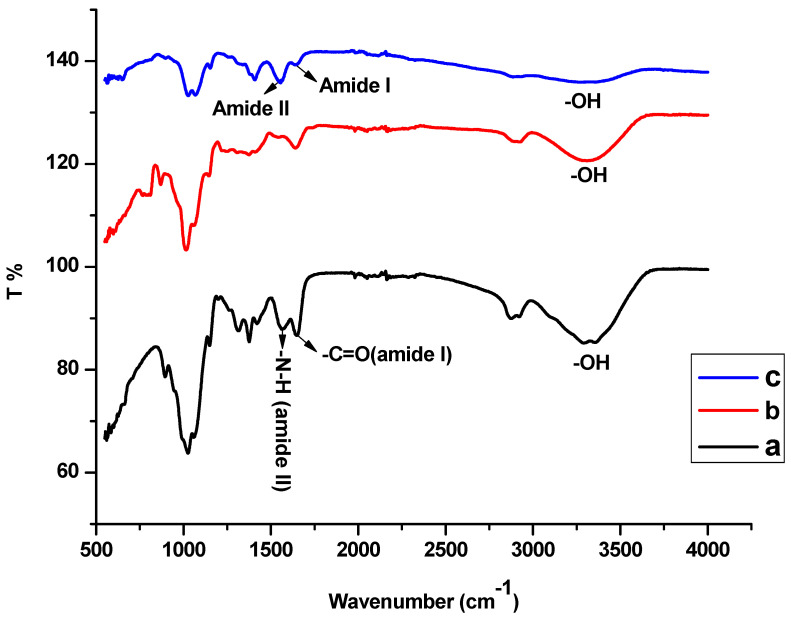
FTIR spectra of (**a**) CS, (**b**) GG, and (**c**) CS-GG polymer blend.

FTIR was used to study the compatibility between chitosan and guar gum. The Chitosan–Guar gum polymeric network structure showed effective blending of the two polymers through the intramolecular and intermolecular hydrogen bonding (Figure 1). The FTIR spectrum of pure chitosan in Figure 2 shows an O-H stretching at 3325.81 cm^−1^, indicating the presence of water, and peaks at 2873.25 cm^−1^ and 1374.02 cm^−1^ corresponding to -CH and -CN stretching, respectively. Furthermore, 1561.99 cm^−1^ -NH deformation (amide II band) from the secondary amine group and 1649.97 cm^−1^ -C=O stretching (amide I band) from the primary amino group were observed. Pure guar gum exhibited characteristic peaks at 3312.59 cm^−1^, 2900.47, and 1010.88 cm^−1^, corresponding to water molecule -OH, -CH, and -CH_2_ group stretching, respectively. The symmetrical deformation of CH_2_ around the 1371 cm^−1^ region was also observed.

A shift in the amine groups to a lower frequency and a decrease in intensity in the polymer blend were observed, confirming the compatibility between chitosan and guar gum. This might be attributed to the hydrogen bonding between the -OH groups of guar gum and -OH and -NH_2_ groups in CS [36].

**Figure 3:**
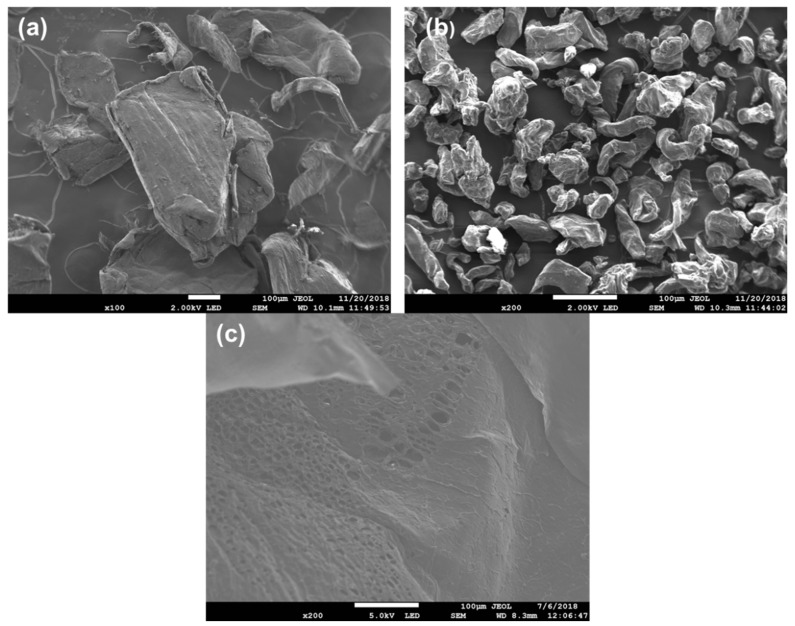
SEM spectra of (**a**) CS, (**b**) GG, and (**c**) CS-GG polymer blend.

**Figure 4:**
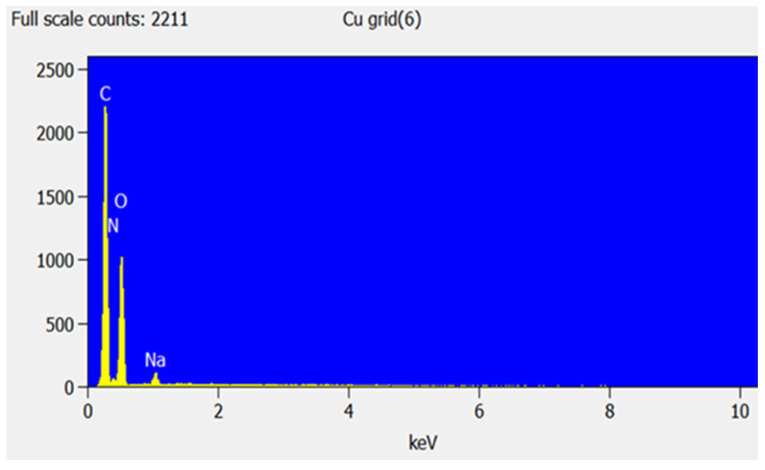
EDS spectra of CS-GG polymer blend.

SEM-EDS was used to study the surface morphology of CS, GG, the CS-GG blend, and the chemical composition of the blend, as shown in Figure 3. From SEM, it was observed that chitosan has an oval pleated shape particle, while guar gum shows a nodule-like shape. However, blending the two showed a remarkable change in the surface morphology of the material, resulting in a porous material with a rough surface. The EDS spectra in Figure 4 confirmed the N, O, C, and Na elements in the polymer blend. The Na element is due to the NaCl solution used when adjusting the pH during the preparation of the chitosan solution.

**Figure 5:**
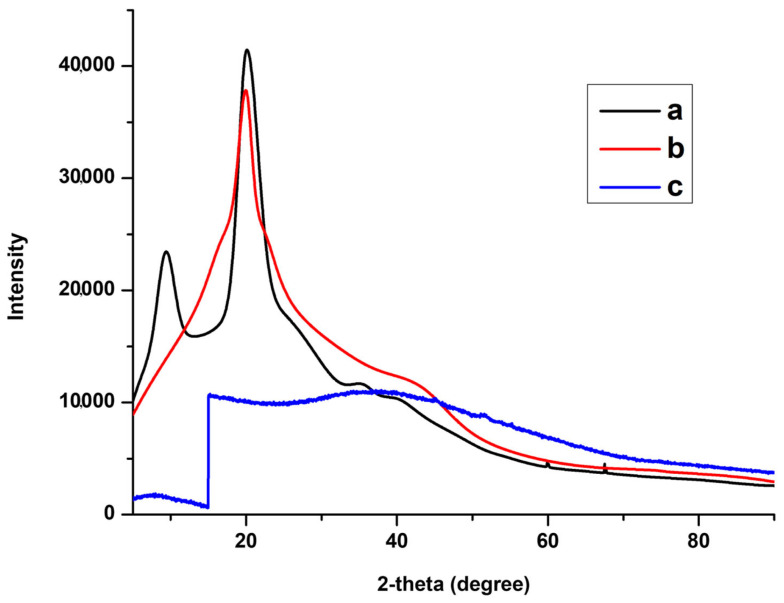
XRD spectra of (**a**) CS, (**b**) GG, and (**c**) CS-GG polymer blend.

The XRD patterns of CS, GG, and the CS-GG blend are shown in Figure 5. The XRD pattern of chitosan showed two semi-crystalline sharp peaks at 2Ɵ = 9.44 and 20.12, which correspond to the (020) and (110) reflections, respectively [15]. Guar gum showed a semi-crystalline peak at 2Ɵ = 19.95. The semi-crystalline structure utterly disappeared in both chitosan and guar gum, resulting in the obtained polymer blend comprising an amorphous pattern. The disappearance of the semi-crystalline structure might be attributed to the formation of hydrogen bonding between the chitosan and guar gum functional groups.

**Figure 6:**
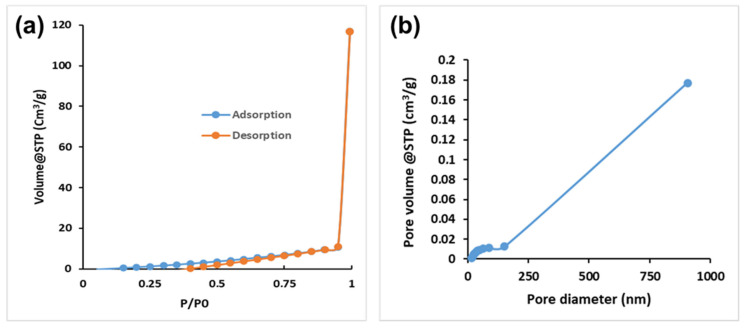
(**a**) N_2_ adsorption–desorption isotherms and (**b**) pore size distribution of CS-GG polymer blend.

Table 1 shows the specific CS-GG pore surface area, pore volume, and pore diameter characteristics. Therefore, CS-GG’s specific pore surface area, volume, and diameter were found to be 11.843 (m^2^/g), 0.184 (cm^2^/g), and 17.072 nm, correspondingly. The mean pore diameter measurement of 17.072 indicates that CS-GG has a mesoporous structure, with pores ranging from 2.0 nm to 50 nm, according to IUPAC. Figure 6 shows the BJH pore width distribution and N_2_ adsorption–desorption isotherms for CS-GG. Based on the results, the CS-GG N_2_ adsorption–desorption isotherm could be categorized as Type-IV, thus demonstrating that a sizable portion of their structure is mesoporous [37,38].

### 3.2. Adsorption Studies

#### 3.2.1. Effect of pH on Hg^2+^ Ion Removal by CS-GG Polymer Blend

**Figure 7:**
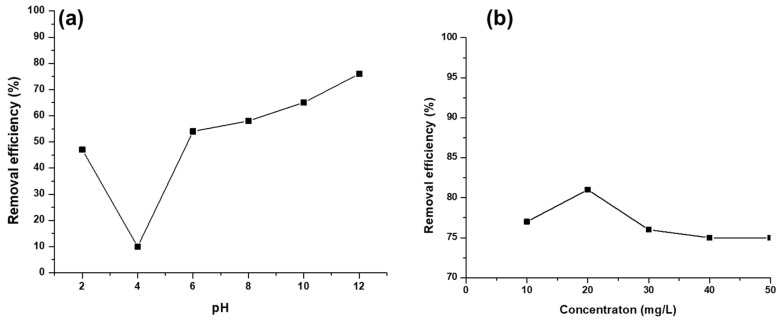
(**a**) Effect of pH and (**b**) effect of initial concentration on the adsorption of Hg^2+^ on the CS-GG polymer blend.

The pH of the solution influences the degree of the sorbent ionization, concentration of counter ions, metal speciation, and surface charge of the adsorbent. Thus, pH is an important parameter in the adsorption process [39,40]. The effect of pH on the adsorption of Hg^2+^ ions was explored in the pH range of 2–12 (Figure 7a), whilst other parameters remained constant. It was observed that as the pH was increased from 2 to 4, there was a great decrease in the removal efficiency of mercury ions. The decrease in the removal efficiency might be attributed to the electrostatic repulsion of protonated functional groups in the polymer blend and the mercury ions, as well as the competition of active sites between the mercury ions and hydronium ions (H^+^) [39]. However, as the pH further increased beyond 4, it showed an increase in the removal efficiency up to pH 12. The increase might be due to the electrostatic attraction and the lone pairs of electrons on the amine and hydroxyl groups, which coordinate with mercury ions, hence forming a readily stable complex. For further studies, pH 12 was selected.

#### 3.2.2. Effect of Initial Metal Ion Concentration

The effect of initial concentration was varied from 10 to 50 mg/L. In Figure 7b, it was observed that mercury removal efficiency increased with an increase in mercury ion concentration from 10 to 20 mg/L. The increase in removal efficiency is due to an increase in ions, thus leading to a higher probability of collision between the mercury ions and the adsorbent [41]. A further increase in initial concentration led to a decrease in Hg^2+^ removal efficiency for CS-GG, then became constant at 40 mg/L. The optimum parameter selected was 20 mg/L.

#### 3.2.3. Effect of Adsorbent Dosage

**Figure 8:**
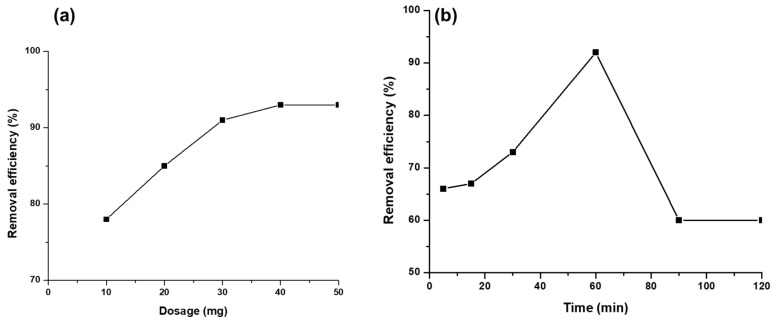
(**a**) Effect of adsorbent dosage and (**b**) effect of contact time on the adsorption of Hg^2+^ on the CS-GG polymer blend.

Figure 8a shows the effect of adsorbent dosage (mg/L) on the removal of Hg^2+^ ions. To study the efficiency of adsorbent dose on the removal of mercury ions, 10, 20, 30, 40, and 50 mg of the CS-GG blend were individually added to the mercury solutions. The results showed an increase in removal efficiency as the adsorbent dosage increased from 10 to 40 mg, eventually reaching equilibrium. This might be due to the increase in active sites, resulting in high efficiency, until the adsorption sites became saturated [42,43].

#### 3.2.4. Effect of Contact Time

The effect of contact time was studied, ranging from 5 to 90 min. It is observed in Figure 8b that Hg^2+^ adsorption efficiency increases from 66% to 92% with the increase in adsorption time from 5 to 60 min. This is attributed to the increase in metal binding time with vacant adsorption sites. A further increase in time beyond 60 min led to a drastic decrease in adsorption due to the optimum capacity of adsorption sites, reaching equilibrium at 90 min [44].

### 3.3. Adsorption Isotherms

The biggest influence of the adsorbent’s adsorption behavior is its adsorption capacity. The Langmuir and Freundlich isotherm models were used to study the adsorption of Hg^2+^ ions. The Freundlich model depicts adsorption on a heterogeneous surface, whereby the adsorbed ions interact, while the Langmuir model depicts saturation monolayer adsorption of solute molecules on a homogeneous surface, whereby the adsorbed ions do not interact [9].

#### 3.3.1. Langmuir Isotherm Model

The Langmuir model is studied using the following equation [45]:

(4) $$\frac{1}{q_{e}}=\frac{1}{K_{L}q_{max}}\left(\frac{1}{C_{e}}\right)+\frac{1}{q_{max}}$$

where *Ce* is the adsorbate’s equilibrium concentration (mg/L), $q_{max}$ is the maximal adsorption capacity (mg/g), $K_{L}$ is the Langmuir adsorption constant (L/mg), and *q_e_* is the quantity of adsorbed metal ions at equilibrium (mg/g).

**Figure 9:**
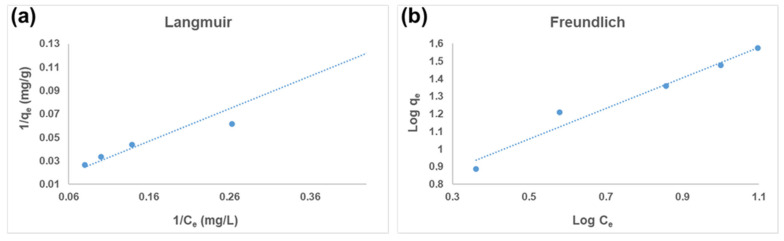
(**a**) Langmuir isotherm and (**b**) Freundlich isotherm for adsorption of Hg^2+^ ions on the CS-GG blend (C_0_ = 20 mg/L, dose = 40 mg, contact time = 120 min, and pH = 12).

Figure 9a shows a linear plot 1/*q_e_* versus 1/*Ce* with a correlation coefficient of R^2^ = 0.9631. The R^2^ shows that the adsorption of Hg^2+^ on the CS-GG blend seems to be less applicable to the Langmuir model (Table 2).

#### 3.3.2. Freundlich Isotherm Model

The Freundlich model is studied using the following equation [46]:

(5) $$\log q_{e}=\frac{1}{n}\log C_{e}+\log K_{F}$$

where *n* and $K_{F}$ (mg/g) are Freundlich constants that relate to the adsorption intensity and multilayer adsorption capacity, correspondingly.

With an R^2^ of 0.9663, Freundlich’s model demonstrated better agreement with the experimental data (Figure 9b). Therefore, the Freundlich model is most suitable for the removal of Hg^2+^ ions on the CS-GG blend compared to the Langmuir model. This suggests a multilayered process involving both chemical and physical adsorption interactions. The heterogeneous and rough surface of the adsorbent likely contributes to this process, as supported by the SEM findings [47].

### 3.4. Kinetics Model Analysis

Two kinetic models, namely pseudo-first-order and pseudo-second-order kinetic models, were used to explain the adsorption of Hg^2+^ on the CS-GG blend.

**Figure 10:**
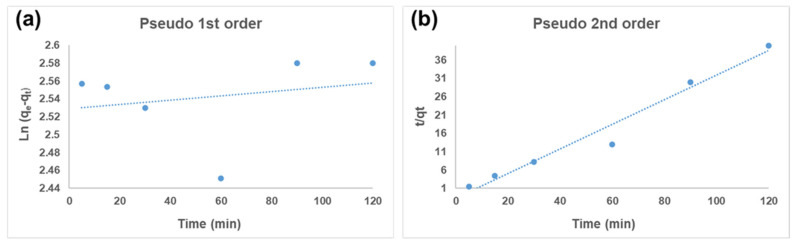
(**a**) Pseudo-first-order and (**b**) pseudo-second-order for adsorption of Hg^2+^ ions on the CS-GG blend (C_0_ = 20 mg/L, dose = 40 mg, contact time = 60 min, and pH = 12).

The pseudo-first-order equation [48] is expressed as follows:

(6) $$\ln(q_{e}-q_{t})=\ln q_{e}-k_{1}t$$

where *k_1_* is the pseudo-first-order rate constant for the adsorption process (min^−1^), and *q_e_* and *q_t_* represent the quantities of Hg^2+^ adsorbed (mg/L) on the CS-GG blend at equilibrium and at a time *t* (min), respectively. A very low correlation coefficient (R^2^ = 0.0507) was obtained from the plot of *ln (qe* − *qt)* versus *t*, displayed in Figure 10a. This suggests that the CS-GG blend’s Hg^2+^ ion adsorption could not adhere to pseudo-first-order kinetics.

The pseudo-second-order equation is expressed as follows [49]:

(7) $$\frac{t}{q_{t}}=\frac{1}{\left(k_{2}{qe}^{2}\right)}+\frac{t}{q_{e}}$$

where *K_2_* represents the pseudo-second-order kinetic model’s rate constant (min^−1^). Figure 10b shows Hg^2+^ ion adsorption on the CS-GG blend. The plot of (*t/q_t_*) versus (*t*) shows a straight line, with a high value correlation coefficient (R^2^) of 0.9687 (Table 3). This suggests that the Hg^2+^ ion adsorption on the CS-GG blend agreed with the pseudo-second-order kinetic model.

### 3.5. Influence of Co-Existing Ions

**Figure 11:**
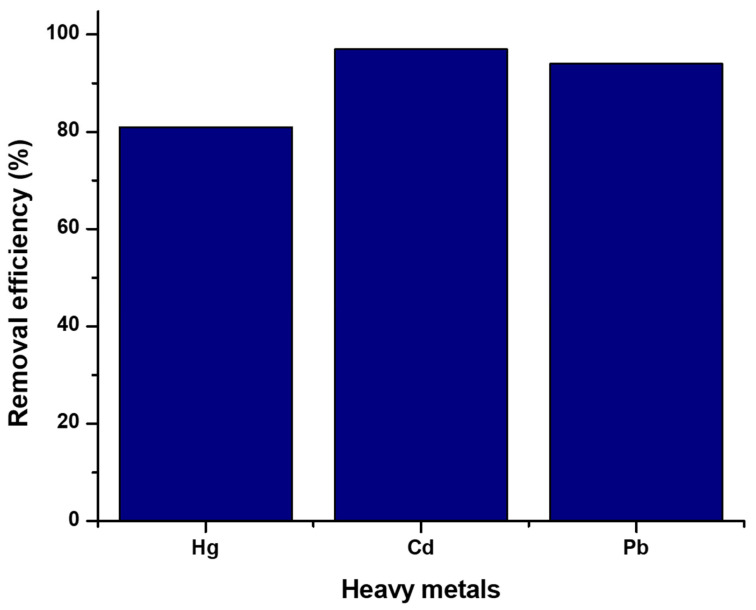
Effect of competing ions in aqueous solution on the removal of Hg^2+^ ions by the CS-GG blend.

The co-existing ions were employed to evaluate the blend’s selectivity for the removal of mercury ions in the presence of Cd^2+^ and Pb^2+^ co-metal ions. The removal of mercury ions was slightly affected in the presence of the two co-metals, where it followed the Cd^2+^ ˃ Pb^2+^ ˃ Hg^2+^ (97; 94; 81) sequence, as shown in Figure 11. This might be attributed to the difference in the ionic radii [50]. It might also be due to Cd^2+^ and Pb^2+^ having higher correlation abilities with the functional groups of the CS-GG blend compared to Hg^2+^ [51].

### 3.6. Comparison of Hg^2+^ Ion Adsorption on CS-GG Blend and Other Adsorbents

A comparative study on the adsorption performance of Hg^2+^ ions on the CS-GG blend to other adsorbents in the literature was investigated (Table 4). It can be seen that the adsorption capacity of the Chitosan–Guar gum blend is incredibly significant compared to other adsorbents reported at lower pH. This shows that the Chitosan–Guar gum blend is an efficient adsorbent for the removal of mercury ions from water in basic media.

### 3.7. Adsorption Mechanism from FTIR

**Figure 12:**
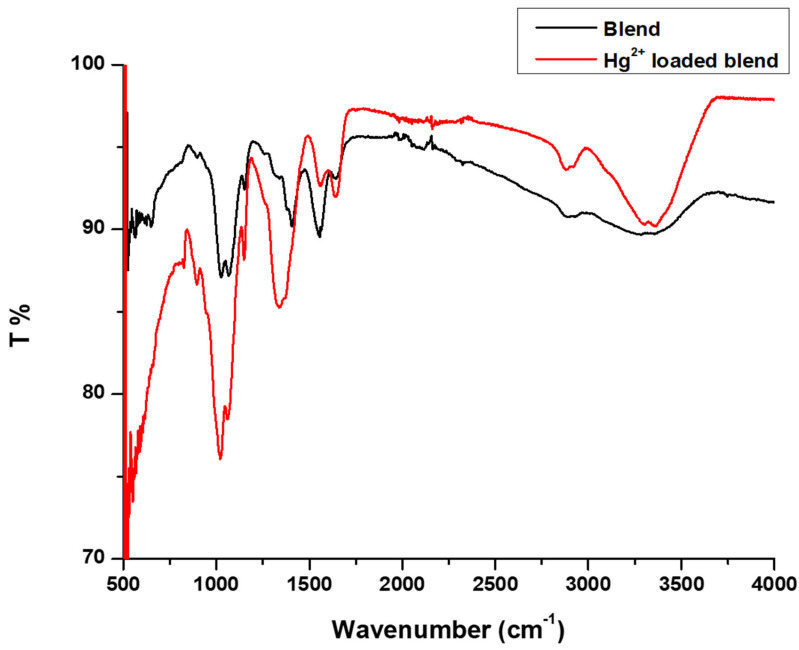
FTIR spectra of CS-GG polymer blend and Hg^2+^ loaded CS-GG polymer blend.

To understand the adsorption mechanism of Hg^2+^ ions on the CS-GG blend, FTIR was used to study and compare the changes in spectra before and after the adsorption of Hg^2+^ ions (Figure 12, Table 5).

(I) The -OH, amide I, and amide II absorption bands of the Hg^2+^ loaded CS-GG blend shifted to a higher frequency.
(II) The Hg^2+^ loaded CS-GG blend OH stretching band showed an increase in intensity and narrowness.
(III) There was an increase and reduction in the intensity of amide I and amide II bands in the Hg^2+^ loaded CS-GG blend, respectively.

**Figure 13:**
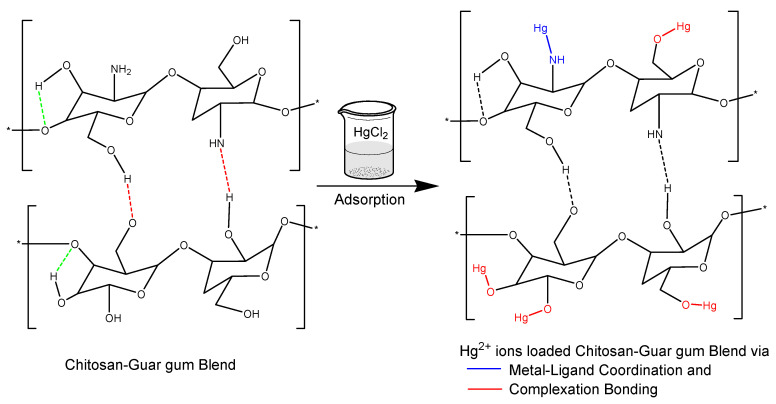
Proposed schematic adsorption mechanism of Hg^2+^ ions on the Chitosan–Guar gum blend. * indicates repeating polymer units.

This proves that there was, indeed, an interaction between the CS-GG blend and the Hg^2+^ ions involved. The interaction could arise to metal–ligand coordination and complexation among the mercury ions and the amine and hydroxyl functional groups on the CS-GG blend (Figure 13).

### 3.8. Desorption and Regeneration Cycles

**Figure 14:**
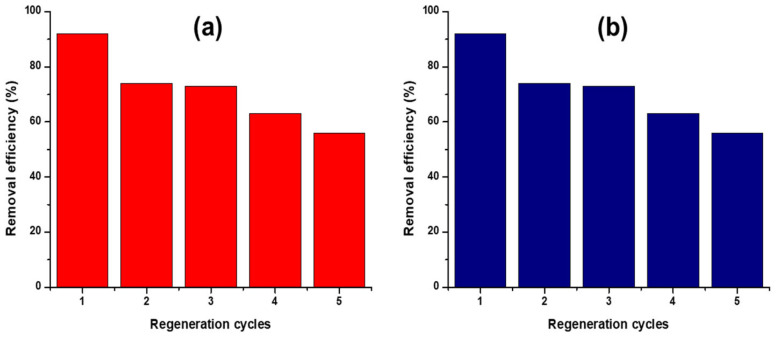
Reusability of the adsorbent for the adsorption of Hg^2+^ ions using (**a**) EDTA and (**b**) NaOH.

The reusability of the CS-GG blend was tested for economic and environmental reasons. Two different desorbing agents were investigated (0.1 M EDTA and NaOH). EDTA is a strong chelating agent for many metal cations. Therefore, it can replace the active site groups on the solid adsorbent with Hg^2+^ ions, forming a very stable complex [55]. The adsorption and desorption processes were repeated for five cycles. From the results, it was observed that the removal efficiency of the CS-GG blend decreased with an increase in several cycles (Figure 14, Table 6), with the removal efficiency on the 5th cycle reaching 56% and 50% using EDTA and NaOH, respectively.

## 4. Conclusions

A novel Chitosan–Guar gum blend was successfully prepared by solution blending for the removal of mercury ions from an aqueous solution. The FTIR confirmed that the blending of chitosan and guar gum was through the hydrogen bonding between the hydroxyl (-OH) group of GG and the hydroxyl and amine (-OH, -NH_2_) groups of CS. The XRD pattern and SEM micrograph conformed to an amorphous and a porous structure of the polymer blend, respectively. The CS-GG polymer blend has proven to be a promising and affordable candidate for the removal of mercury ions in water. The blend showed a high removal efficiency of 92% at pH 12 in 60 min. This shows and confirms that mercury ions were stable at a higher pH due to Au^3+^. Studies on desorption and regeneration also showed that it can be reused for several cycles, making it a practical and sustainable adsorbent. The adsorption behavior was in accord with the pseudo-second-order kinetic model and Freundlich adsorption isotherm model, demonstrating multilayer adsorption with chemisorption interactions.

## Figures and Tables

**Table 1:** CS-GG’s surface area and porosity.

Parameters	Values
Pore surface area (m^2^/g)	11.843
Pore volume (cm^3^/g)	0.184
Pore diameter (nm)	17.072

**Table 2:** Isotherm constants for adsorption of Hg^2+^ ions from aqueous solution.

Langmuir Model		Freundlich Model	
q_max_ (mg/g)	$K_{L}$ (L/mg)	1/n	$K_{F}$ (mg/g)
R^2^		R^2^	
370.37	0.0097	0.8689	4.2072
0.9631		0.9663	

**Table 3:** Kinetic parameters for the adsorption of Hg^2+^ ions.

Kinetic Model	Constants		
Pseudo-first-order	q_e_ (mg/g) 12.5422	K_1_ (min^−1^) 0.0002	R^2^ 0.0507
Pseudo-second-order	q_e_ (mg/g) 2.9806	K2 (g/mg/min) 0.1898	R^2^ 0.9687

**Table 4:** Maximum adsorption capacity value of Hg^2+^ ions on the CS-GG polymer blend compared with other adsorbents.

Adsorbent	pH	Adsorption Capacity (mg/g)	References
Diatomite-chitosan	3	195.7	[52]
Chitosan-g-poly granular hydrogel	5.5	365.55	[3]
Chitosan/carboxymethyl guar gum biopolymer	6	151.51	[53]
Hydrous manganese oxides onto acylamino and hydroxyl functionalized hydrogel	8	131.2	[54]
Chitosan–Guar gum polymer blend	12	370.37	This work

**Table 5:** FTIR characteristic peaks of CS-GG and Hg^2+^ loaded CS-GG polymer blend.

CS-GG Polymer Blend	Hg^2+^ Loaded CS-GG Polymer Blend
3293.49—O-H stretching	3320.87—O-H stretching
2901.75—C-H stretching	2894.91—C-H stretching
1637.59—C=O stretching (amide I band)	1638.47—C=O stretching (amide I band)
1548.63—N-H stretching (amide II band)	1555.47—N-H stretching (amide II band)
1406.65—C-N stretching	1339.93—C-N stretching
1042.28—C-O-C stretching	1021.75—C-O-C stretching

**Table 6:** Reusability of the adsorbent for the adsorption of Hg^2+^ ions.

Desorbing Agent	Adsorption %				
	1st cycle	2nd cycle	3rd cycle	4th cycle	5th cycle
EDTA	92	74	73	63	56
NaOH	92	70	68	60	50

## Data Availability

The data presented in this study are available on request from the corresponding author due to legal or ethical reasons.
